# Surface versus Bulk Currents and Ionic Space-Charge Effects in CsPbBr_3_ Single Crystals

**DOI:** 10.1021/acs.jpclett.2c00804

**Published:** 2022-04-25

**Authors:** Osbel Almora, Gebhard J. Matt, Albert These, Andrii Kanak, Ievgen Levchuk, Shreetu Shrestha, Andres Osvet, Christoph J. Brabec, Germà Garcia-Belmonte

**Affiliations:** † Institute of Advanced Materials (INAM), Universitat Jaume I, 12006 Castelló, Spain; ‡ Institute of Materials for Electronics and Energy technologies (i-MEET), Friedrich-Alexander Universität Erlangen-Nürnberg, 91058 Erlangen, Germany; § Department of General Chemistry and Chemistry of Materials, Yuriy Fedkovych Chernivtsi National University, 2, Kotsyubynsky St., 58012 Chernivtsi, Ukraine; ∥ Erlangen Graduate School in Advanced Optical Technologies (SAOT), Friedrich-Alexander Universität Erlangen-Nürnberg, 91052 Erlangen, Germany

## Abstract

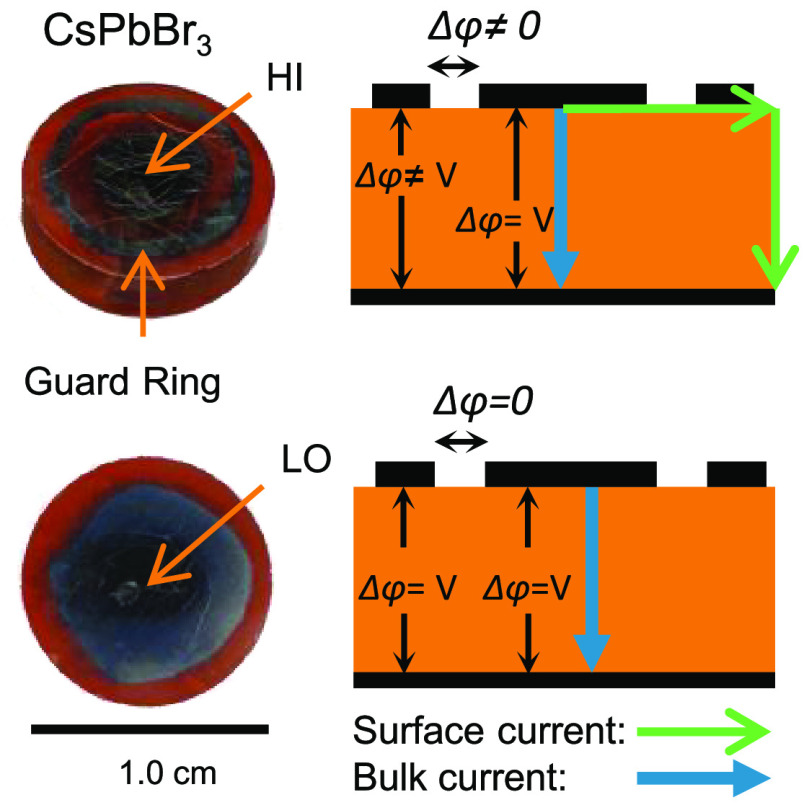

CsPbBr_3_ single crystals have potential for application in ionizing-radiation detection devices due to their optimal optoelectronic properties. Yet, their mixed ionic–electronic conductivity produces instability and hysteretic artifacts hindering the long-term device operation. Herein, we report an electrical characterization of CsPbBr_3_ single crystals operating up to the time scale of hours. Our fast time-of-flight measurements reveal bulk mobilities of 13–26 cm^2^ V^–1^ s^–1^ with a negative voltage bias dependency. By means of a guard ring (GR) configuration, we separate bulk and surface mobilities showing significant qualitative and quantitative transport differences. Our experiments of current transients and impedance spectroscopy indicate the formation of several regimes of space-charge-limited current (SCLC) associated with mechanisms similar to the Poole–Frenkel ionized-trap-assisted transport. We show that the ionic-SCLC seems to be an operational mode in this lead halide perovskite, despite the fact that experiments can be designed where the contribution of mobile ions to transport is negligible.

The all-inorganic cesium lead tribromide perovskite (CsPbBr_3_) is an attractive material with several perspective applications in photovoltaics,^1^ photodetectors,^2,3^ and light emitting devices^4,5^ due to its optoelectronic properties^6,7^ and various fabrication methods.^8−10^ Particularly, in the field of ionizing energy detection, many studies have been focused on thin film approaches,^9,11^ yet the use of millimeter-thick single crystals has gained more attention recently.^2,3^ Importantly, most of the reported studies in the literature focus on the optical properties and the fast optoelectronic response from the material and the devices, respectively. However, little is discussed about the current density–voltage characteristics (*J–V*) which typically show nonlinearities and bias-sweep-direction-dependent features (hysteresis)^12^ due to the dual electronic–ionic conductivity of these materials,^13,14^ similar to other organo-metal-halide perovskite thin film devices.^15,16^

In dark conditions, the *J–V* curve is typically analyzed in symmetrically contacted samples to identify different transport regimes. Commonly, the ohmic region (*J ∝ V*) and a transition toward the classic mobility regime of space-charge-limited current (SCLC) are found, where the latter follows the Mott–Gourney law^17,18^
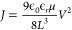
1Here *L* is the distance between electrodes, *ϵ0* is the vacuum permittivity, *ϵ*_*r*_ is the dielectric constant (∼40 for CsPbBr_3_),^19^*L* is the distance between electrodes, and *μ* is the mobility of the electronic charge carriers. Moreover, a trap-filled-limited region can occur when *J ∝ V*^*n*^, with *n* > 2,^18,20^ and a ballistic-like voltage-dependent mobility (BVM)^21^ regime can also take place when *n* = 3/2. The BVM regime resembles the Child–Langmuir law^22,23^ in terms of the current trend above the threshold voltage *V*_0_ with a law as
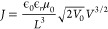
2

However, unlike the classic ballistic transport, where no mobility can be defined, the BVM model describes the case of a field-dependent mobility  with threshold value *μ_0_* at *V_0_*. This modifies the classic definition of the drift velocity as a function of the electric field *ξ*. Phenomenologically, the BVM regime constitutes a particular case of Poole–Frenkel ionized-trap-assisted transport^24−26^ with a field-dependent distribution of charge carriers.^21^ Recently, the BVM model has been found to describe the long-term kinetics of electronic transient currents in MAPbBr_3_ single crystals.^27^

Over all SCLC types, few reports^28,29^ have identified these regimes with clarity in CsPbBr_3_ single crystals. More often, the Mott–Gourney law is misused in ohmic-like saturation regimes at higher voltages (see Table S1 in the Supporting Information).^30−32^ Likewise, hysteresis control and reproducibility of the results are often missing. Notably, in thin film devices the current pathways are mostly throughout the grain boundaries and/or the bulk material. Accordingly, the charge carriers effectively travel a distance *L* from one electrode to the other. However, in thick single crystals the charge carriers can travel a distance *L* across the bulk material with either a bulk resistivity *ρ*_*b*_ or a distance *L*_*s*_ throughout the surface (single-crystal grain boundaries) with surface resistivity *ρ*_*s*_.

In classic semiconductors, the ohmic surface electronic currents *J*_*s*_ can be significantly higher than bulk currents *J*_*b*_ when *ρ_s_L_s_ < ρ_b_L* due to the presence of significant unintentional doping at the surface. However, the question arises on how the transport evolves regarding the mixed electronic–ionic conductivity of lead halide perovskites. Depending on the polarization history of a sample, a field-induced redistribution of free mobile ions dominates the long-term evolution of the electrical response. Still, it is not clear whether the mobile ions are mostly distributed along the surface, along the bulk, or at the vicinity of the electrodes.^33,34^

The matter of avoiding surface leakage currents can be tackled by using the guard ring (GR) configuration. In this arrangement, not only is the sample geometry prevented from affecting the *J–V* characteristics, but one can also discern between surface and bulk contributions. The active electrode is surrounded by a closed metallic connection line on top of the surface (the guard ring) with the same electrostatic potential *ϕ* of the electrode. Consequently, no electric current will flow between the top electrode (HI) and the GR, as depicted in Figure S1. The current between the GR and the bottom (LO) contact is driven by a unity gain buffer with a high input impedance. With this arrangement, just the direct current through the bulk of the crystal is measured, since the local electric potential difference between the HI and the GR is zero (*Δϕ* = 0). Notably, in Figure S1*L* is the distance between the HI and the LO and *L_s_> L* is an alternative pathway for the charge carriers to travel without the GR. This approach has successfully been applied in X-/γ-ray detectors based on, for example, CdTe,^35,36^ CdZnTe,^37,38^ Si,^39−43^ and SiC.^44^ More recently, Wei et al.^45^ showed that using GR reduces the crystal surface leakage current and device dark current in CH_3_NH_3_PbBr_3–*x*_Cl_*x*_-based γ-ray detectors.

In this work, the surface and bulk contributions to the current density throughout 1–3-mm-thick CsPbBr_3_ single crystals are quantified using the GR configuration. The samples are tested via long-time current transients and impedance spectroscopy (IS) for illustrating the time scale of the electronic and ionic kinetics. We show that the long-time ionic relaxation kinetics and many of the often-found SCLC regimes are mostly surface phenomena. We estimate values for ion-affected electronic mobilities by analyzing the steady-state currents and the leakage low frequency resistances. Furthermore, we propose prebiasing experiments that allow the estimation of the transient concentration of mobile ions toward the electrodes creating transient depletion layer capacitances.

*Field-Dependent Mobility from Time-of-Flight Experiments*. For a start, the ToF measurements are presented in Figure 1 characterizing the transport of bulk-photogenerated charge carriers across the bulk of the sample. This is a condition close to equilibrium since the bias pulses have periods up to hundreds of microseconds. The transit time between electrodes was between 15 and 50 μs, resulting in mobility values ranging 13–26 cm^2^ V^–1^ s^–1^ with a seemly negative bias dependency as *μ ∝ V^–1/2^* (solid line in Figure 1b) in the electric field (*ξ*) range 100–900 V cm^–1^. Among lead halide perovskites, the negative field dependency of mobility (*∂μ/∂ξ* < 0) has been previously reported in CH_3_NH_3_PbBr_3_ for a similar field range,^46^ whereas the positive case (*∂μ/∂ξ* > 0) has been predicted by classical molecular dynamics simulations of halide ionic mobilities in CH_3_NH_3_PbI_3_ for *ξ* > 1 MV cm^–1^.^47^ The field dependency of the charge carrier mobility is an intensively reported and modeled behavior in inorganic and organic semiconductors, as summarized in Table S2. Either by hopping^48−50^ or by the trap-mediated Poole–Frenkel^51,52^ effect, several Monte Carlo simulations suggest that the negative field dependency of the mobility may appear in some limit cases of transport and morphology properties.

**Figure 1:**
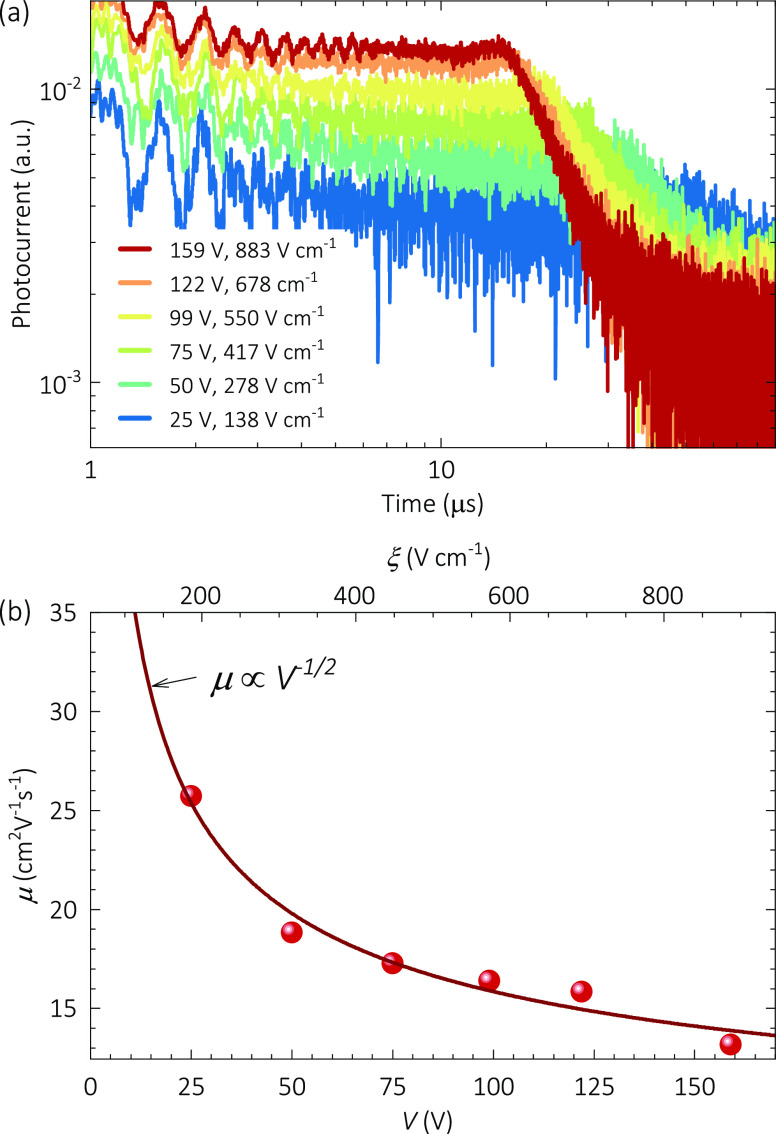
Time-of-flight measurements of a CsPbBr_3_ single crystal: (a) photocurrent transients and (b) corresponding mobilities as a function of bias. The solid line in part b corresponds to an allometric fitting, following the trend of the BVM model,^21^ as indicated. No GR connection was used for this experiment.

Notably, the experimental trend *μ ∝ V^–1/2^* is in agreement with the BVM model,^21^ if *ξ* = *V*/*L* (top axis in Figure 1b) is considered. This may relate to mobile ions which modify the electronic transport properties upon biasing, even for the fast pulse perturbation in the ToF experiment. More importantly, the bias-dependent mobility implies major changes in the way space charges distribute, which subsequently modifies the current. This suggests that one can no longer define a “true” absolute mobility for the material at a given temperature. Instead, a mobility value can be taken for a given condition or as an average effective value within voltage and temperature ranges. Moreover, the question arises on the influence of ionic effects when comparing bulk and surface transport.

*Hysteresis and Long-Term Current Transients with and without a Guard Ring*. Typical *J–V* characteristics were measured for several crystals, in different polarization routines. In every experiment, the surface current values (without a GR) exceeded those of the bulk (with a GR), as shown in Figure S2. For some extreme cases, not only the current was reduced with the GR connection, but also the hysteresis, as presented in Figure 2a. Interestingly, the hysteretic *J–V* curves without a GR behave like a sequence of regimes as *J ∝ V^n^*, where the power 1 < *n* < 3.2 varies with the bias range and polarization time, which could be understood in terms of the formalism of SCLC, as a consequence of an effective field-dependent mobility with *μ ∝ ξ^n–2^*.^21^ On the other hand, the bulk related currents measured with the GR show ohmic behavior with a specific resistivity ρ = 0.7 GΩ cm, in agreement with reports by Stoumpos et al.^53^

**Figure 2:**
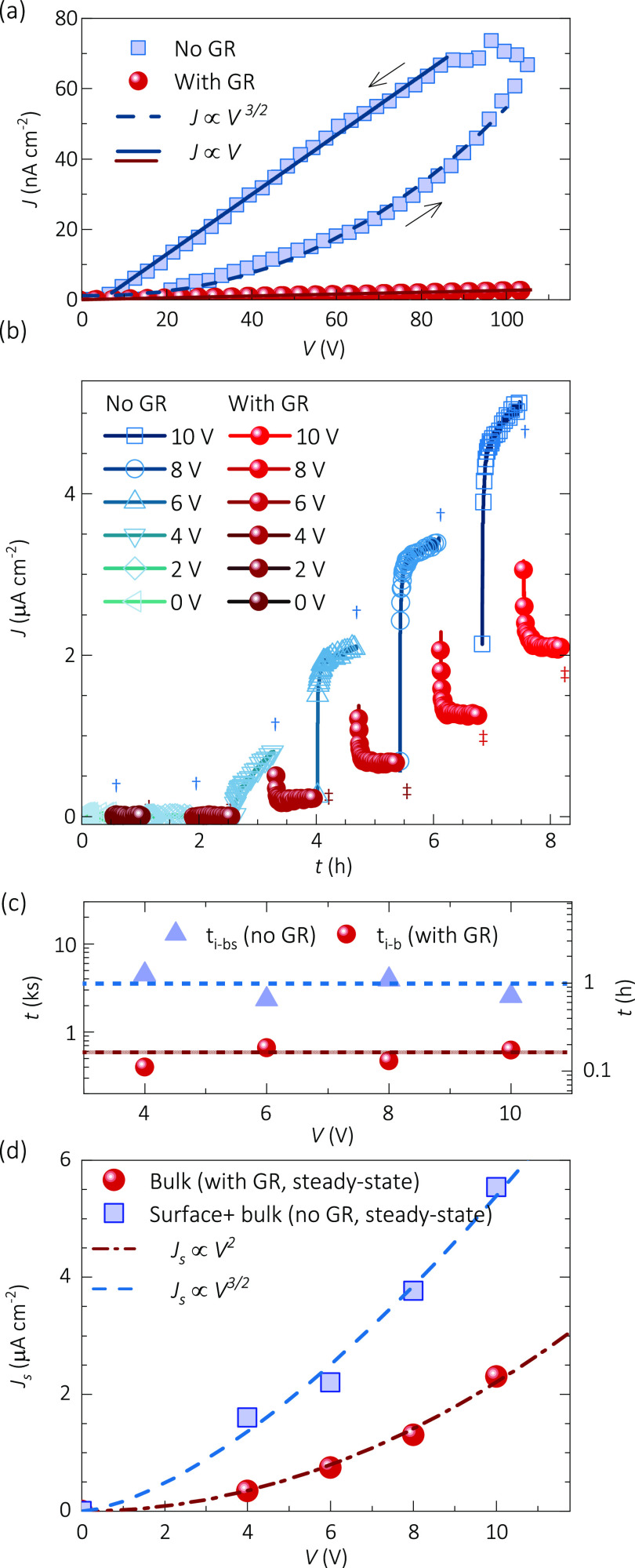
Dark current–voltage characteristics of CsPbBr_3_ single crystals with and without a guard ring: (a) continuous bias sweep at 150 mV s^–1^, (b) stepwise sequence with a 2.5 ks delay at each voltage bias, (c) characteristic relaxation times, and (d) steady-state saturation currents for current transients in part b from exponential fittings (see Figure S3). The solid, dashed, and dash-point lines in parts a and d indicate allometric fittings as indicated. The ‡ and † symbols in part b indicate when the IS measurements were performed with and without guard rings, respectively (see Figure 3 and Figure S4).

In a subsequent experiment, the long-term current evolution was explored at different DC biases, as indicated in the sequence of Figure 2b. At each voltage value (between the HI and the LO) the current is monitored, first during 2.5 ks without a GR and, subsequently, when the GR is connected and the current is sensed for another 2.5 ks. The procedure was continuously repeated up to 10 V. In this bias range and time window, the bulk currents (with a GR) contributed only 20–40% of the total bulk + surface currents (without a GR), meaning that surface currents are contributing 60–80%. The current transients (Figure 2b) were fitted to exponential relaxation models (see Figure S3) whose characteristic times are summarized in Figure 2c. Neglecting faster processes, the ionic bulk kinetics seems to achieve steady-state current after *τ_i-b_*∼ 500 s (with a GR), regardless of the applied voltage. Differently, without a GR, the bulk + surface currents show ionic relaxations with *τ_i-bs_* > 3 ks.

The saturation steady-state currents (*J*_*s*_) are always larger than those in typical voltage sweeps, and the resulting trends may indicate different SCLC mechanisms with or without a GR, as shown in Figure 2d. Across the bulk (with a GR), a typical Mott–Gurney law^17^ of the mobility regime of SCLC could be assumed from the behavior *J* ∝ *V*^2^, resulting in the mobility *μ_GR_* = 53 cm^2^ V^–1^ s^–1^. This bulk mobility is between two and three times larger than that extracted from the ToF experiment, partly because of the different field range (see extrapolation in Figure 1b) but mostly due to the conductivity and space-charge modification during the prebiasing period. On the other hand, the surface contribution (no GR) introduces an important component *J ∝ V^3/2^*. This may relate to a BVM regime of SCLC,^21^ from which a higher mobility of *μ_0,nGR_* = 414 cm^2^ V^–1^ s^–1^ can be estimated. This presumably surface mobility surpasses all the estimations for bulk mobility (*μ_nGR_* ≫ *μ_GR_* > *μ*) with a value somehow closer to previous reports in nonstabilized *J–V* curves.^30^

*Impedance Spectroscopy with and without a Guard Ring*. Simultaneously to the chronoamperometry experiment, the IS spectra with and without a GR were measured after the current stabilization at each voltage bias (see marks ‡ and † in Figure 2b). The full set of data in the Nyquist representation and the fitting parameters can be found in Figure S4 and Table S3, respectively, and an illustrative plot is shown in Figure 3a along with the equivalent circuit model used to simulate the spectra. There, *R_0_* is the equilibrium resistance (under 0 V DC bias), which is kept constant during all the simulations.

**Figure 3:**
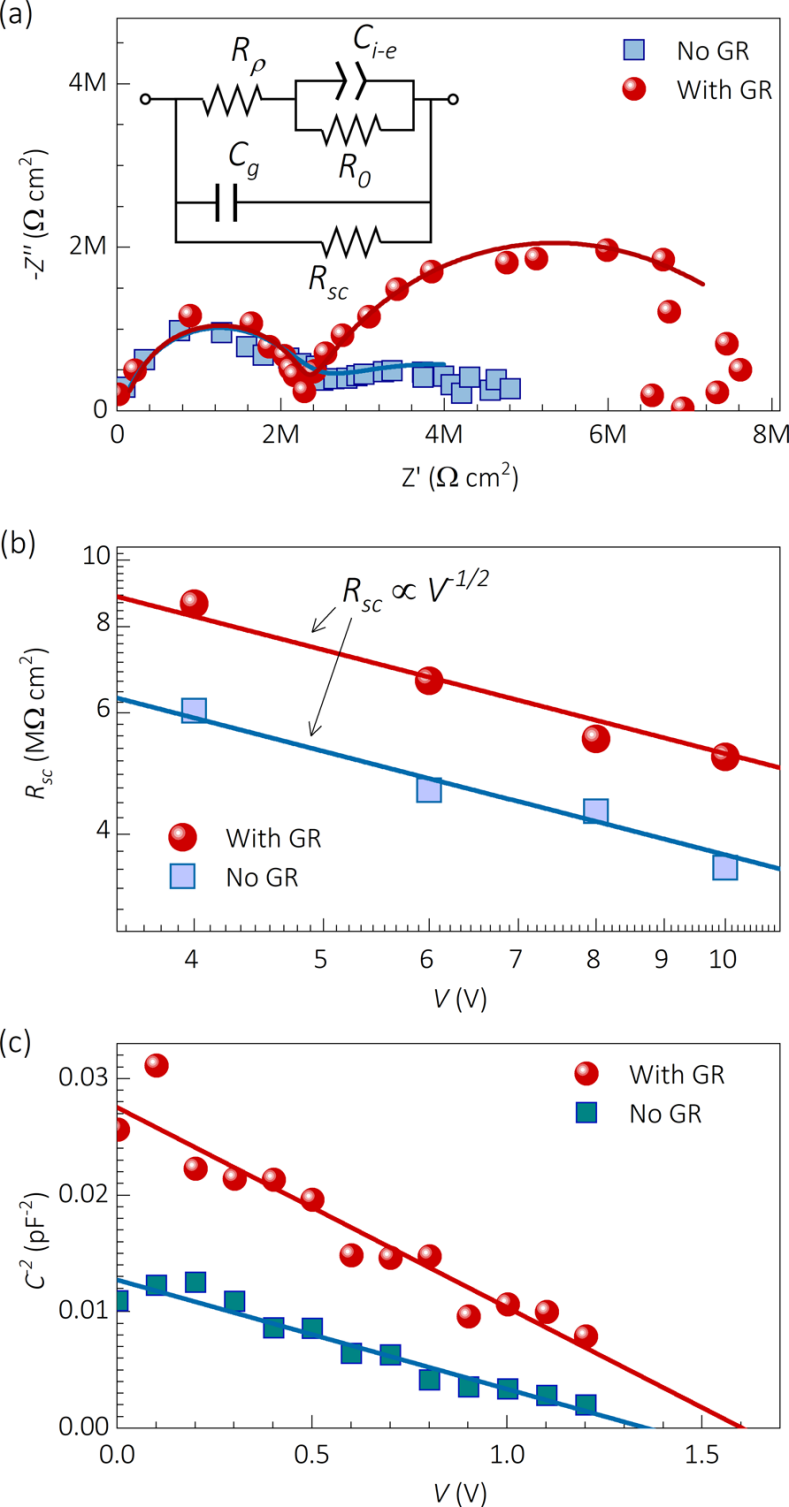
Impedance spectroscopy characterization of 3-mm-thick CsPbBr_3_ single crystals with and without a guard ring: (a) impedance Nyquist plot at 6 V; (b) resistances resulting from fitting to the equivalent circuit model inset in part a; and (c) Mott–Schottky plots at 100 kHz after 2.5 h at 10 V of prebiasing.

Toward higher frequencies, the dielectric capacitance *C*_*g*_ and a coupled seemly ohmic resistance *R*_*ρ*_ form a first arc. This high frequency section of the spectrum behaves independently of whether the GR is connected or not. Here the dielectric capacitance is practically the geometrical contribution *C_g_ ≈ ε_0_ε_r_/L* of the sample, although some minor contributions of depletion layer capacitance (*C*_*dl*_) cannot be fully discarded. The applied DC voltage and the GR connection make no significant variation on *R*_*ρ*_ and *C*_*g*_. Accordingly, these high frequency parameters are associated with the transport in the bulk.

Toward lower frequencies, a second arc appears (see Figure 3a) which is simulated with an ionic–electronic capacitance *C_i–e_* in addition to the space-charge-related resistance *R_sc_*. Below *V_0_* = 4 V of DC bias, the impedance is mostly defined by *R*_*0*_ and *C*_*g*_, and then a transition occurs above *V*_*0*_ (see Figure S4). Afterward, the ratio between *R*_*sc*_ and *R*_*ρ*_ (both ≪ *R*_*0*_) with *C*_*i*–*e*_ defines the bias- and surface/bulk-transport properties. Above *V_0_*, the *R*_*sc*_ results are larger with a GR than that without a GR; that is, the more available the surface is for transport, the smaller the resistance. Furthermore, either with or without a GR, the trend *R_sc_ ∝ V^–1/2^* is observed, which agrees with the BVM regime of SCLC. Applying the differential resistance *R* = *(dJ/dV)^−1^* definition to the current of eq 2 and neglecting *∂μ/∂V* components in the derivative, one can find^21^
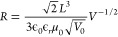
3

Equation 3 was used to fit the *R*_*sc*_ behavior (solid lines in Figure 3b), resulting in mobilities of *μ_0,GR_* = 98 cm^2^ V^–1^ s^–1^ and *μ_0,nGR_* = 137 cm^2^ V^–1^ s^–1^, with and without a GR, respectively. Once again, our mobility parametrization shows smaller values for the experiment with a GR (bulk-related) in comparison to that without a GR (bulk + surface-related). Interestingly, the fact that the BVM regime of SCLC relates to the low frequency-related part of the measured IS spectra suggests the main contribution of the mobile ions to the charge density profile.

Notably, the discrepancy of the mobility values between the DC (transient *J*_*s,*_ previous section) and the AC (*R*_*sc*_ from IS) experiments is related to two main factors. First, the GR connection for the IS measurement is set for the DC bias, while the AC perturbation includes both bulk and surface currents. This is probably why the SCLC mode of *R*_*sc*_ is the BVM with a GR, instead of the Mott–Gurney law. Second, *R*_*sc*_ includes both the surface and bulk contributions to the space-charge effect, meaning an overestimation of the resistance. Separating the *R*_*sc*_ components is not a trivial task; thus, the mobilities from the IS measurements should be considered as minimum values.

Further evidence of ionic-mediated space-charge regions can be found by analyzing the high frequency dielectric capacitance in different prebiasing experiments. For instance, using the Mott–Schottky (MS) analysis^54,55^ after periods of prebiasing, one can obtain capacitance steps depending on the sign of the previous poling (see Figure S5). Moreover, if a sufficiently long prebias is applied, e.g. 2.5 h at 10 V, clear MS plots are obtained such as those of Figure 3b when the bias sweep is fast enough so that the space charge does not relax back to equilibrium before the measurement is done.

In the MS formalism, the depletion layer of width *W*_*dl*_ decreases with the external bias and the depletion layer capacitance is expressed as , where *N* is the concentration of mobile ions creating the transient built-in voltage *V_bi_*. Notably, the application of the MS analysis to the experiment of Figure 3b requires the depletion layer width to be smaller than the device thickness (*W_dl_= ϵ_0_ϵ_r_/C_dl_≤ L*) in order to be measurable. Accordingly, a capacitance correction factor of 3.16 was considered accounting for possible smaller parasitic capacitances in series. Subsequently, the concentration values resulted as *N_GR_* = 6.7 × 10^8^ cm^–3^ and *N_nGR_* = 9.3 × 10^8^ cm^–3^, with and without a GR, respectively. The larger transient doping concentration without a GR suggests that more ions are able to accumulate across the surface during the time window of the experiment. Once more, the surface is shown to favor the ion migration in these materials, in agreement with the previous experiments, as summarized in Table 1.

**Table 1:** Estimated Parameters for the Comparison between Surface and Bulk Transport in the Studied CsPbBr_3_ Single Crystals^a^

Use of guard ring	*μ* from ToF [cm^2^ V^–1^ s^–1^]	*μ* or *μ_0_* from *J*_*s*_ [cm^2^ V^–1^ s^–1^]	*μ_0_* from *R*_*sc*_ [cm^2^ V^–1^ s^–1^]	*N* from MS [cm^–3^]
Yes	−	54	98	6.7 × 10^8^
No	13–26	413	137	9.3 × 10^8^

a Excepting the ToF bulk mobility measurement, it is only when the GR is connected that the parameter values relate exclusively to the bulk.

In summary, the electrical response of millimeter-thick CsPbBr_3_ single crystals was characterized via fast ToF measurements, long-term DC current transients, and IS measurements with/without the use of GR connections. Our findings suggest a strong dependency of the electronic mobility on the space-charge distribution of mobile ions upon biasing. In the classic sense, the bulk mobility from the ToF results in between 13 and 26 cm^2^ V^–1^ s^–1^ with a negative voltage bias trend as *μ ∝ V^–1/2^*. We strongly recommend these as the most appropriate values to use for device simulations and material comparisons. Nevertheless, the apparent field dependency of the mobility should be considered. The origin of this behavior is most likely due to a conductivity modification, such as the Poole–Frenkel ionized-trap-assisted transport,^24−26^ which cannot be experimentally discerned from the mobility. Accordingly, different mobility values can be found depending on the polarization routines which create different regimes of SCLC. Importantly, even though one can design experiments to discard the mobile-ion-formed space charges, the ionic-mediated SCLC seems to be the operational mode.

In the time scale of hours, the current showed a slow relaxation upon biasing that is shortened to a few minutes when a GR connection is used, suggesting that surface and grain boundary defects are the main pathway for ionic migration. The steady-state values of the current for the explored bias range are associated with SCLC mobility regimes. In the time scale up to seconds, after hours of relaxations, the IS measurements showed how the surface transport is related to the slower ionic component. In addition, transient depletion layer capacitance experiments were shown to create built-in fields due to accumulation of mobile ions toward the surfaces with concentrations in the order of 10^8^ cm^–3^.

## Experimental Section

The studied CsPbBr_3_ crystal samples were fabricated with the Bridgman–Stockbarger^56,57^ method, following the procedure reported by Stoumpos et al.^53^ with the specific conditions described in Section S4 of the Supporting Information. The resulting single crystals had cylindric shapes with ∼1.1 cm diameter and 0.3 cm thickness, as illustrated in Figures S1 and S6. The morphology, stoichiometry, and crystallinity of the sample were checked via electron scanning microscope (SEM) images and energy-dispersive X-ray (EDX) and X-ray diffraction (XRD) spectra, as presented in Figure S6. The optical properties of the samples are summarized in Figure S7, where the absorbance, transmittance, photoluminescence (PL), and Tauc plot spectra illustrate the optical band gap of 2.2 eV and two PL peaks at 561 nm (2.2 eV) and 523 nm (2.38 eV). Particularly, the double PL peak emission has been reported by several authors^9,53,58−61^ and associated with the contribution from localized or free excitons^59^ and recombination involving Br vacancy centers.^60^ The samples were contacted with sputtered platinum. Pt was chosen as contact material due to the inert nature of this metal and the fact that the high work function provides a hole selective contact. Time-of-flight (ToF) measurements were conducted with a nanosecond Nd:YAG laser in the setup described by Shrestha et al.^62^ for a range from 25 to 159 V of pulsed biases.

For the measurement of dark continuous current (DC) mode *J–V* curves, a Keithley 236 was utilized at room conditions. Several protocols of impedance spectroscopy (IS) characterizations were used in this work by means of a bipotentiostat PGSTAT302N-FRA32M, from Metrohm Hispania AUTOLAB. The alternating current (AC) mode voltage perturbation was 150 mV. The GR connection was via an AUTOLAB’s BA unit, which provides a second working electrode. The temperature was controlled with a microprobe system with a Peltier device heating and a cooling sample stage, from Nextron.

## Supporting Information Available

Summary of literature reports on SCLC in CsPbBr_3_ and field-dependent mobility in semiconductors; experimental details on fabrication and basic optoelectronic material characterization; current–voltage, chronoamperometries, and impedance spectroscopy data and fittings (PDF)
