# Supplementary material for: Surface versus Bulk Currents and Ionic Space-Charge Effects in CsPbBr3 Single Crystals
Source: J Phys Chem Lett. 2022 Apr 25;13(17):3824–30. doi: 10.1021/acs.jpclett.2c00804 (PMC9082610; doi:10.1021/acs.jpclett.2c00804)
Supplement: Supplementary file 1 — jz2c00804_si_001.pdf [file jz2c00804_si_001.pdf]

Supporting information:

Surface versus Bulk Currents and Ionic Space-Charge Effects in $CsPbBr_3$ Single Crystals

Osbel Almora[1,2,*], Gebhard J. Matt[3,*], Albert These[2,3], Andrii Kanak[4], Ievgen Levchuk[3], Shreetu Shrestha[3], Andres Osvet[3], Christoph J. Brabec[2,3], Germà Garcia-Belmonte[1,*]

[1] *Institute of Advanced Materials (INAM), Universitat Jaume I, 12006 Castelló, Spain*
[2] *Erlangen Graduate School in Advanced Optical Technologies (SAOT), Friedrich-Alexander Universität Erlangen-Nürnberg, 91052 Erlangen, Germany*
[3] *Institute of Materials for Electronics and Energy technologies (i-MEET), Friedrich-Alexander Universität Erlangen-Nürnberg, 91058 Erlangen, Germany*
[4]*Yuriy Fedkovych Chernivtsi National University, Department of General Chemistry and Chemistry* of *Materials*, 2, *Kotsyubynsky St., 58012 Chernivtsi, Ukraine*
** corresponding authors*
E-mail addresses: almora@uji.es, gematt@ethz.ch, garciag@uji.es

S1. Introduction

Table S1: Summary on literature studied on dark current voltage characteristics and electrical properties of symmetrically contacted $CsPbBr_3$ single crystals.

Fabrication method	Dark current-voltage characteristic		Sample contacts	Electrical properties	Year [Ref.]
	Regimes	Hyst./ Asym.			
Bridgman	i. Ohmic (0-300 V)	-	$Ag/CsPbBr_3/Ag$ (2.1 mm SC-TC)	ρ =1-343 GΩ cm $m_n=m_p=0.23m_e$	2013[1]
LTSB	-	-	$CP/CsPbBr_3/CP$ (1-0.5 mm SC-TC)	ρ =55-135 MΩ cm ε=38-45	2016[2]
LTSB	i. Ohmic (0-20 V) ii. Trap filled ($J\propto V^5$, 20-133 V) iii. SCLC (133-200 V)	-	$Au/CsPbBr_3/Au$ (1.25 mm SC-TC)	μ_p=11 $cm^2 V^{-1} s^{-1}$ ρ =100 MΩ cm ε=22	2017[3]
LTSB	i. Ohmic (0-6 V) ii. Trap filled ($J\propto V^{3.9}$, 6-15 V) iii. SCLC (15-20 V)	-	$Ti/CsPbBr_3/Ti$ (1.25 mm SC-TC)	μ_n=52 $cm^2 V^{-1} s^{-1}$	2017[3]
Bridgman (modified)	i. Ohmic (0-1 V) ii. Trap filled ($J\propto V^6$, 1-3 V) iii. Ohmic saturation (7-13 V)*	Asym.	$Au/CsPbBr_3/Au$ (1.1 mm SC-TC)	μ_p=2060 $cm^2 V^{-1} s^{-1}$ ε=16	2017[4]
Bridgman (modified)	i. Ohmic (0-1 V) ii. Trap filled ($J\propto V^6$, 1-2 V) iii. Ohmic saturation (3-12 V)*	-	$Ag/C_{60}/CsPbBr_3/C_{60}/A$ g (1.1 mm SC-TC)	μ_n=2240 $cm^2 V^{-1} s^{-1}$	2017[4]
LTSB	i. Ohmic (0-5 V)	-	$Au/CsPbBr_3/Au$ (0.1 mm SC-LC)	-	2017[5]
LTSB	i. Ohmic (0-30V)	Asym.	$Au/CsPbBr_3/Au$ (0.5 mm SC-LC)	ρ =840 MΩ cm	2017[6]
VPEG	i. Ohmic (0-1 V) ii. Trap filled ($J\propto V^{2.5}$, 2-5 V) iii. Ohmic saturation (7-10 V)*	Asym.	$Au/CsPbBr_3/Au$ (50 μm SC-LC)	-	2017[7]
Bridgman	i. Ohmic (±50V)	Asym.	$Au/CsPbBr_3/Au$ (3 mm SC-TC)	-	2018[8]
EDG	ii. Ohmic (±10V)	Asym.	$Au/CsPbBr_3/Au$ (6 mm SC-LC)	-	2018[9]

EDG	i. Ohmic (±10V)	-	$Ag/CsPbBr_3/Ag$ (1 mm SC-TC)	ρ =2-4 TΩ cm	2018[10]
Bridgman	ii. Ohmic (±1V)	Hyst. + Asym.	$Au/CsPbBr_3/Au$ (SC-TC)	ε=40	2019[11]
LTSB	i. Ohmic (0-1V)<br>ii. Trap filled ($J \propto V^{1.2}$, 1-10V)<br>iii. SCLC (10-20V)	-	$Au/CsPbBr_3/Au$ (SC-TC)	ρ =350 MΩ cm<br>μ_p=7.4 $cm^2 V^{-1} s^{-1}$	2020[12]
LTSB	i. Trap filled ($J \propto V^{1.2}$, 1-10V)*<br>ii. SCLC (13-20V)	-	$Ag/BCP/C_{60}/CsPbBr_3/C_{60}/BCP/Ag$ (SC-TC)	μ_n=0.63 $cm^2 V^{-1} s^{-1}$	2020[12]
LTSB	i. Ohmic (1-7V)*	-	$Au/CsPbBr_3/Au$ (0.5 mm SC-TC)	μ_p=1.1 $cm^2 V^{-1} s^{-1}$	2021[13]

Abbreviations: Current density (J), voltage (V), hysteresis (Hyst.), asymmetry (Asym.), single crystal transversally contacted (SC-TC), single crystal laterally contacted (SC-LC), reference (Ref.), low-temperature solution-based (LTSB), solution based inverse temperature crystallization (SB-ITC), vapor-phase epitaxial growth (VPEG), electronic dynamic gradient (EDG), resistivity (ρ), carbon paste (CP), dielectric constant (ε), effective mass for electrons and holes (m_n and m_p, respectively), electron and hole mobilities (μ_n and μ_p, respectively), * incorrectly attributed SCLC.

Table S2: Field-dependent mobility $\mu(\xi)$ models and experiments in the literature.

System	Dependency	Notes	Ref.	
	Mobility increases with the field $(\partial\mu/\partial\xi > 0)$		Theo.	Exp.
Amorphous semiconductors; e.g. PE	$\mu = \mu_0 \frac{\xi_0}{\xi} \sinh\left[\frac{\xi}{\xi_0}\right]$	Hopping transport models: μ_0 and ξ_0 are characteristic mobility and field, respectively, which are functions of the activation energy and the temperature.	14-18	19
Inorganic semiconductors; organic solids with off-diagonal disorder; conjugated polymers; e.g. Cl:a-Se, PPV, PFO, DEASP:PC	$\mu \approx \mu_0 \exp\left[\sqrt{\frac{\xi}{\xi_0}}\right]$	Poole-Frenkel effect behaviour in systems with low disorder; time of flight (ToF) measurements.	20-27	25-33
Inorganic semiconductors; molecularly doped polymers; e.g. GaSe, DEASP:PC	$\mu \approx \mu_0 \exp\left[\left(\frac{\xi}{\xi_0}\right)^m\right]$	ToF and Hall effect measurements; $0 < m < 1$		29, 34
Insulators	$\mu \approx \mu_0 \left(\frac{\xi}{\xi_0}\right)^m$	Analytical model; $m \geq 0$	35-37	
	Mobility decreases with the field $(\partial\mu/\partial\xi < 0)$			
Organic semiconductors; e.g. C_8-BTBT crystalline films	$\mu \approx \mu_0 \left(\frac{\xi}{\xi_0}\right)^{-m}$	ToF measurement; effective-medium approximation (EMA) modelling; case of high field regime for charge carrier hopping transport based on Marcus theory and variable-range hopping theory; $m > 0$	38, 39	40
Hopping transport in organic spatially disordered semiconductors	$\mu = \mu_0 \frac{\left(N_0 + N_t \exp\left[\frac{\Delta E}{k_B T}\right]\right)}{\left(N_0 + f(\xi) N_t \exp\left[\frac{\Delta E}{k_B T}\right]\right)}$; $f(\xi) \approx f_0 \exp\left[\left(\frac{\xi}{\xi_0}\right)^{3/2}\right]$	Analytical model: two energy levels separated ΔE, with concentration N_0 of sites with higher energy, much larger than the concentration N_t of sites with lower energy. $f(\xi)$ is an approximated function of the field where ξ_0 decreases with the increase of the material disorder.	41	

Organic solids with off-diagonal disorder; e.g. PPV	$\mu \approx \mu_0 \exp\left[-\left(\frac{\xi}{\xi_0}\right)^m\right]$	ToF measurements; Monte Carlo simulations for systems of high disorder at intermediate and low field regime; $m > 0$	21	42
Organic semiconductors with correlated Gaussian disorder; π-conjugated polymers; e.g. P3OT, PFO, G1F, P3HT, TPD:PS, DCJTI: PFO, DEASP:PC, P3HT:PCBM, RRPOT	$\mu \approx \mu_0 \exp\left[-\sqrt{\frac{\xi}{\xi_0}}\right]$	ToF, impedance spectroscopy (IS) & charge extraction by linearly increasing voltage (CELIV) measurements; analytical & numerical modelling: case of high field regime.	43-49	50-57
Disordered molecular solids.	$\mu \approx \mu_0 \frac{\xi_0}{\xi} \sinh\left[\frac{\xi}{\xi_1}\right] \exp\left[-\left(\frac{\xi}{\xi_2}\right)^2\right]$	Monte Carlo simulations of high-field hopping mobility	58	

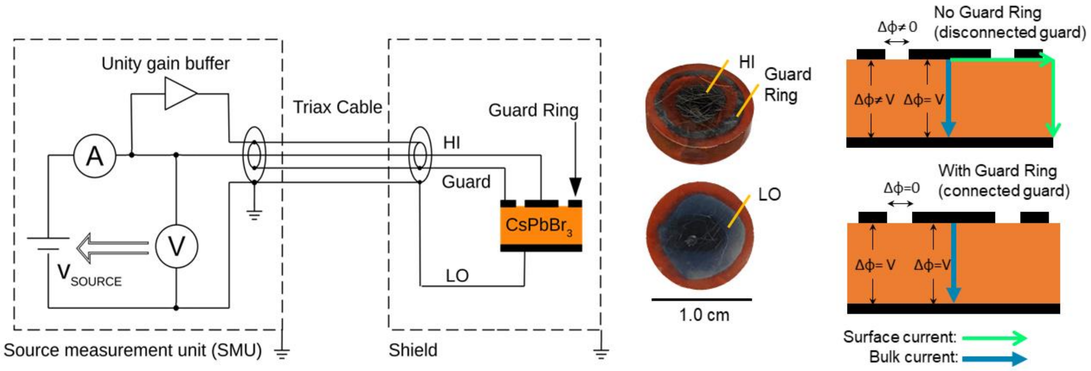

Figure S1: Schemed guard ring connections for evaluation of surface versus bulk current contributions. In the left panel, the circled A and V stand for amperemeter and voltmeter, respectively. The right panel shows front-lateral and rear pictures of one of the studied single crystal $CsPbBr_3$ samples with 1.1 cm of diameter and 3.0 mm of thickness. In the extreme right sketches, V and $\Delta\phi$ are the external applied DC bias and the local difference in electrostatic potential, respectively.

S2. Current-voltage characterization

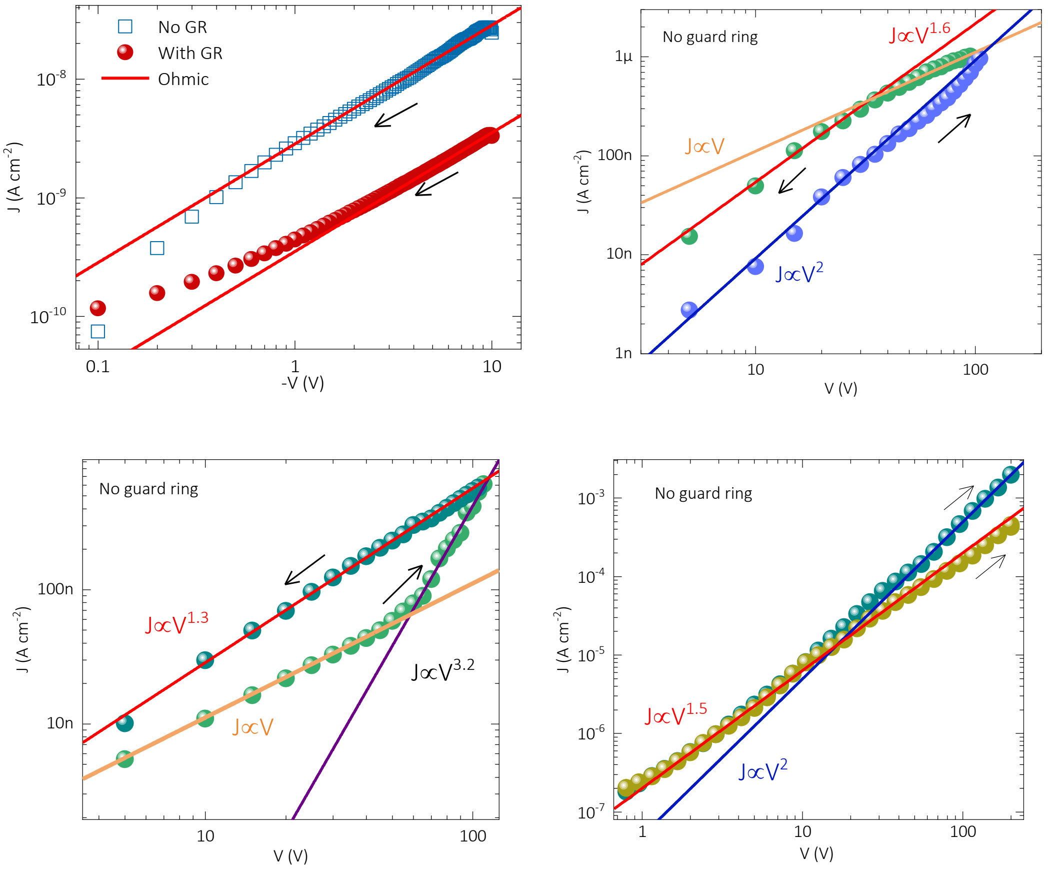

Figure S2: Current density-voltage curves without and with guard ring (GR). The dots are experimental data and the solid lines are fittings to allometric functions as indicated. The arrows indicate the voltage sweep directions, with scan rates in the range 0.05-10 V s^{-1}.

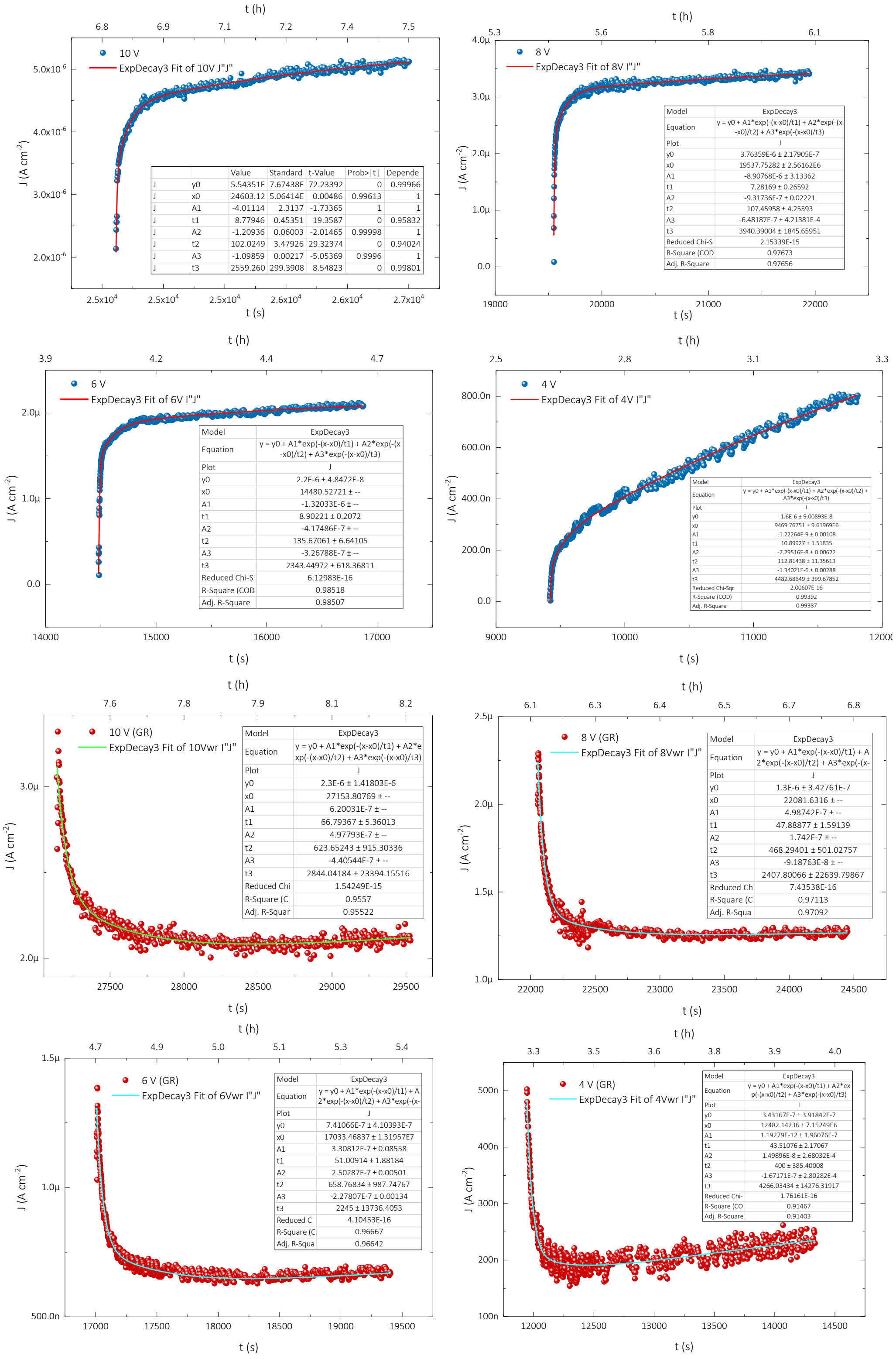

Figure S3: Current transients at different DC biases, without and with guard ring (GR), as indicated. The solid lines are the fitting to exponential relaxation laws whose characteristic times and saturation currents are summarized in Figure 3c,d, respectively.

S3. Impedance spectroscopy characterization

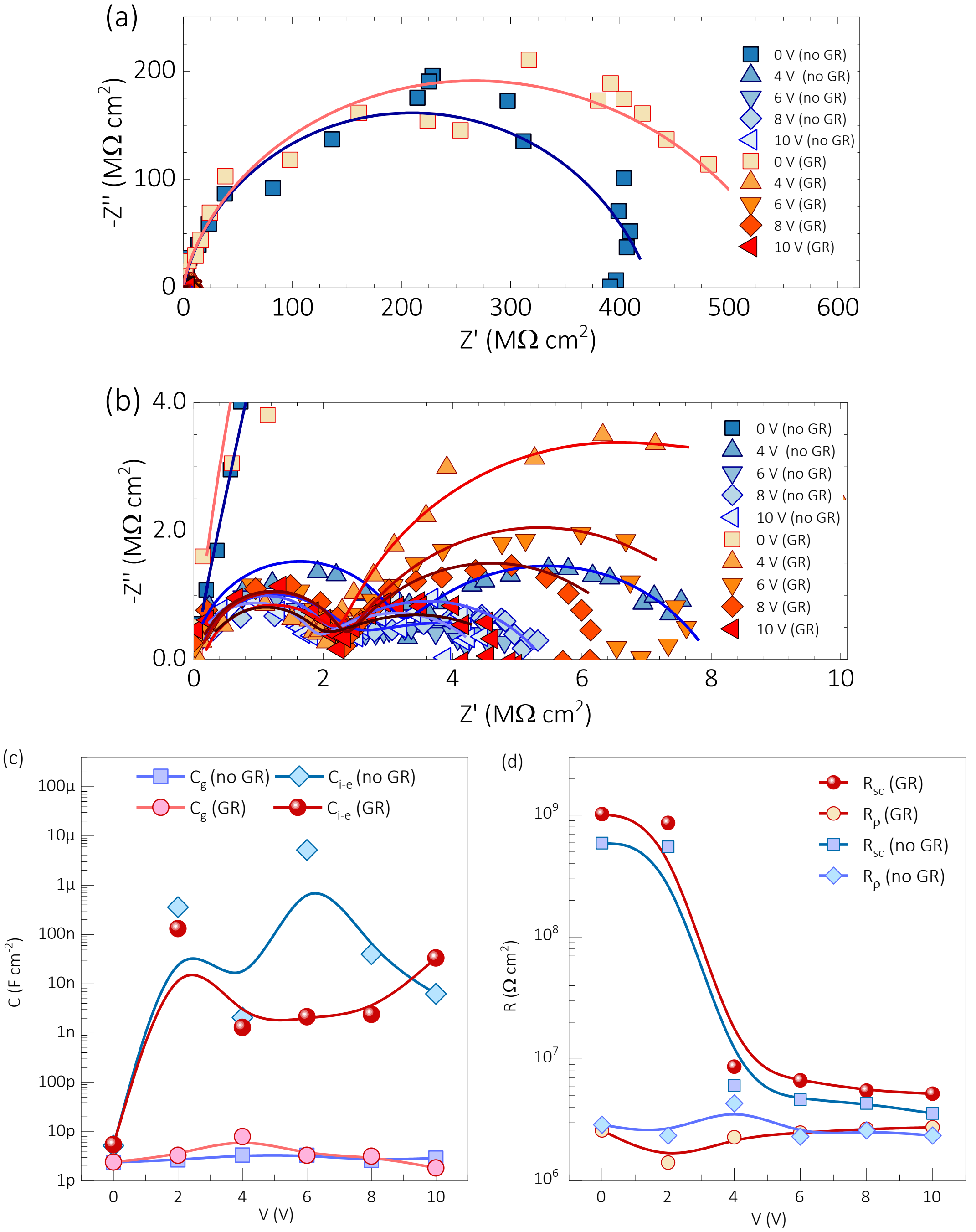

Figure S4: Impedance spectroscopy (a,b) experimental (dots) and simulated (lines) spectra for a 3 mm thick CsPbBr3 single crystal after stabilization of different DC biases (see Figure 3b), with and without guard ring (GR). The simulations are based on the equivalent circuit model sketched in Figure 4a, reporting the behaviors of the (c) capacitances and the (d) resistances, as indicated.

Table S3: Fitting parameters for simulated spectra in Figure S4a,b from the equivalent circuit model of Figure 4a. The effective area was 0.7854 cm^2; p_g and p_{i-e} are the power parameters for the constants phase elements (CPE) used to simulate the capacitances C_g and C_{i-e}, respectively.

V (V)	R_{sc} (Ω)	C_g ($F^{1/pg}$)	p_g	R_0 (Ω)	Ci-e ($F^{1/pi-e}$)	p_{i-e}	R_ρ (Ω)
With guard ring							
0	1.30E9	1.90E-12	1.00	1.00E9	2.90E-11	0.65000	3.30E6
2	1.10E9	2.60E-12	1.00	1.00E9	1.23E-8	0.54000	1.80E6
4	1.10E7	2.89E-11	0.84	1.00E9	1.02E-9	0.85463	2.90E6
6	8.50E6	2.60E-12	1.00	1.00E9	1.48E-9	0.75640	3.17E6
8	7.00E6	2.49E-12	1.00	1.00E9	1.59E-9	0.72947	3.40E6
10	6.60E6	1.44E-12	1.00	1.00E9	5.07E-9	0.49618	3.50E6
No guard ring							
0	7.51E8	1.87E-12	1.00	1.00E9	1.65E-11	0.74615	3.68E6
2	7.00E8	2.10E-12	1.00	1.00E9	2.74E-9	0.17884	3.00E6
4	7.70E6	4.50E-12	0.95	1.00E9	1.39E-9	0.67891	5.50E6
6	5.90E6	2.60E-12	1.00	1.00E9	2.00E-8	0.36034	2.94E6
8	5.50E6	2.06E-12	1.00	1.00E9	5.93E-9	0.51643	3.30E6
10	4.55E6	2.28E-12	1.00	1.00E9	2.67E-9	0.61624	3.00E6

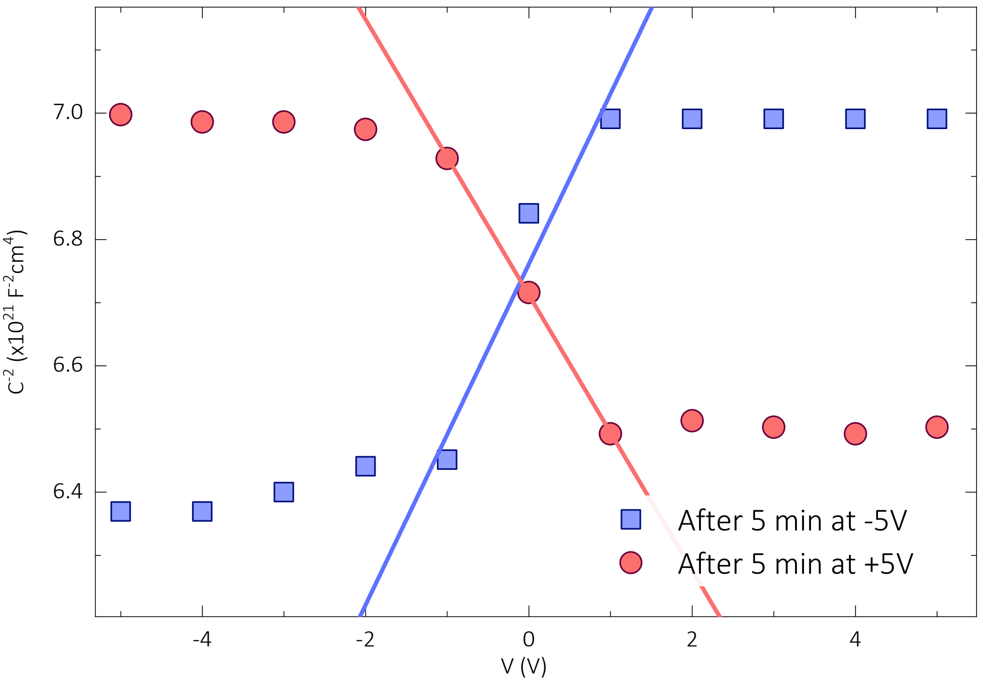

Figure S5: Mott-Schottky (MS) analysis of transient capacitance switching the capacitance sweep direction after a short time pre-bias, as indicated.

S4. Experimental

Fabrication description: $CsPbBr_3$ single crystals were obtained by the Bridgman–Stockbarger method similar as was described by Stoumpos et al.[1]. CsBr (manufactured by Alfa Aesar) and $PbBr_2$ (was obtained by precipitation from KBr and $Pb(NO_3)_2$ water solutions) were stoichiometrically mixed and ground in an agate mortar. During grinding, the mixture changed color from white to deep orange. Then the material was loaded into a quartz ampoule, evacuated to 10^{-4} mbar, and sealed. The ampoule was placed in a three-zone vertical furnace, slowly heated to 585 °C, and held for several hours. Next, the ampoule was lowered into a cold zone of the furnace at a rate of 3 mm/h. After completing the directional crystallization of the melt, the furnace was cooled down to room temperature. Orange-red transparent crystals about 50 mm in length and 11 mm in diameter were grown. The ingots were cut by a wire-cutting machine and polished using Al_2O_3 powder with a 1 and 0.3 µm grain sizes. The Pt contact were sputtered with a JEOL JFC-1200 Fine coater in Argon atmosphere at a pressure of 8 Pa and a sputtering current of 30 mA for 600 s.

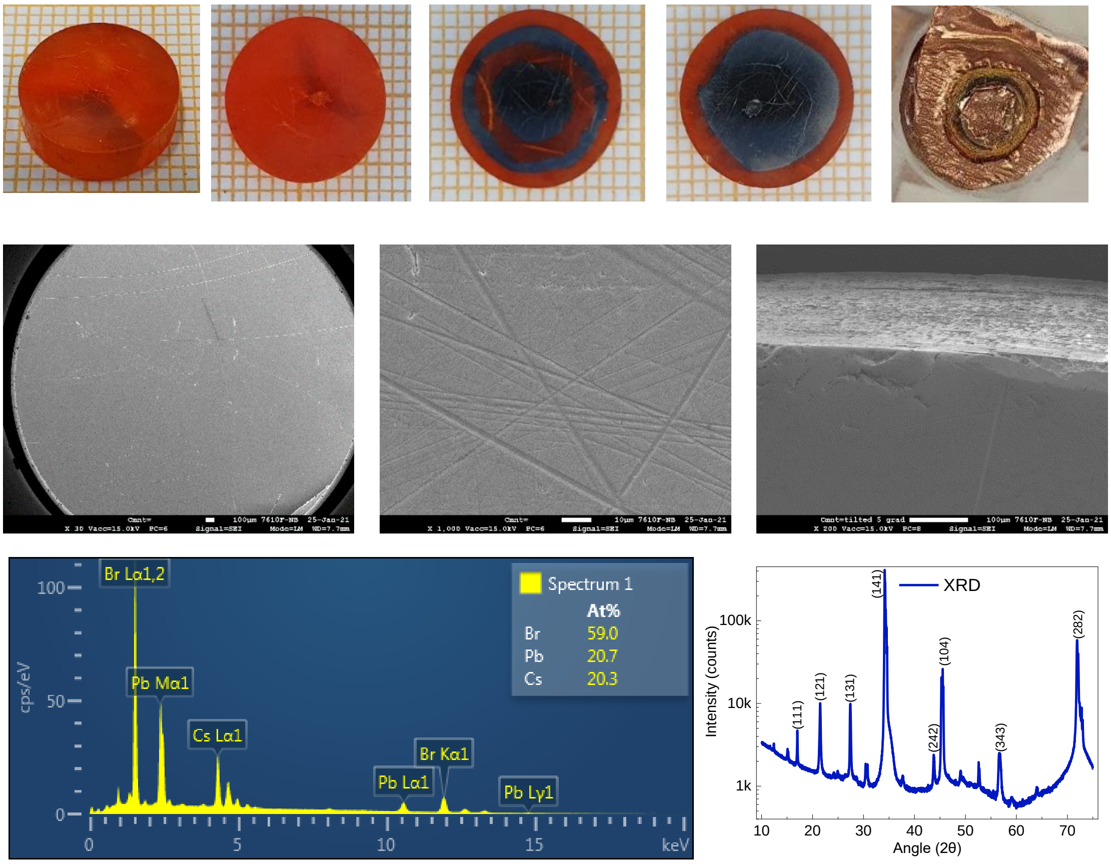

Figure S6: Morphologic characterization. In the top row, pictures before and after sputtering Pt contacts illustrate the size of the samples and contacts on top of millimetre-grid paper. In the top right, the conductive tape connections are shown, including the scratched space between the inner contact and the guard ring for preventing mask failures during Pt evaporation. In the middle row, scanning electron microscope (SEM) images show the surface topography. In the bottom row, the energy-dispersive X-ray (EDX, left) and X-ray diffraction (XRD, right) spectra illustrate stoichiometry and crystallographic features of the samples.

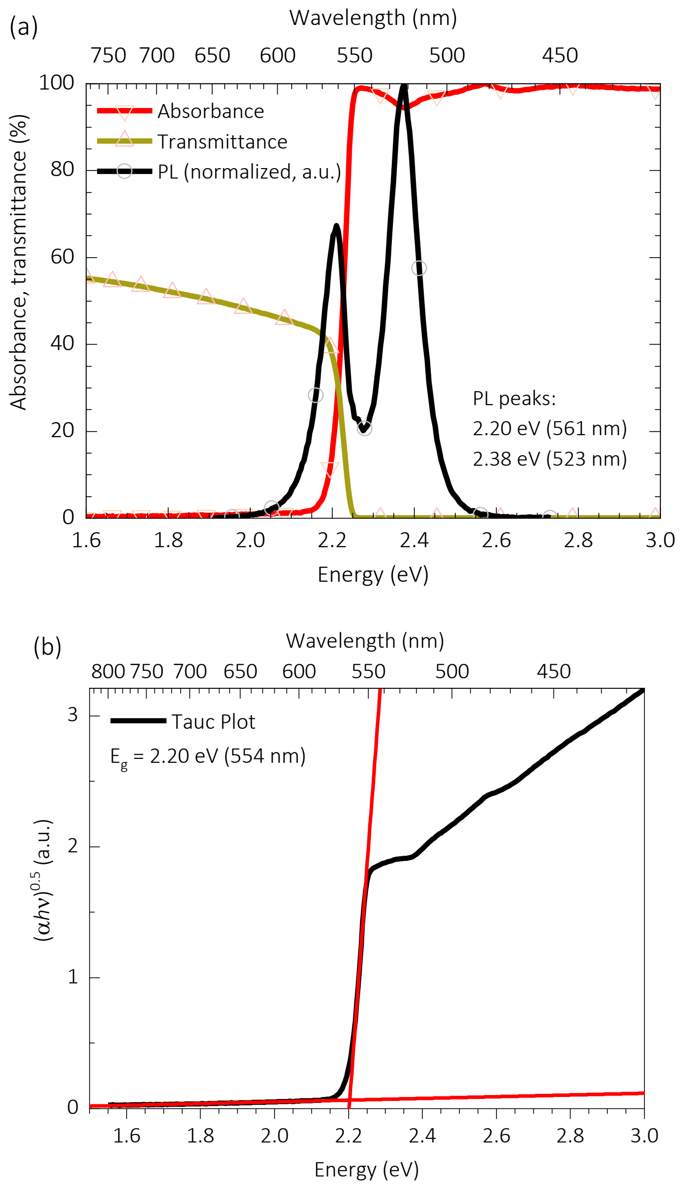

Figure S7: Optical characterization: (a) absorbance, transmittance, photoluminescence (PL) and (b) Tauc Plot from the absorption coefficient (α) spectrum, assuming direct bandgap. In (b) h is the Planck's constant, v is the photon frequency and E_g is the bandgap energy. The lower energy PL peak have been reported by several researchers around 2.20±0.07 eV,[5, 59, 60] which matches the bandgap. This suggests a direct band-to-band recombination. The higher energy PL peak has also been reported by several studies around 2.38±0.07 eV[4, 10, 13, 61, 62], which suggests a major radiative recombination between states within the bands. Moreover, the simultaneous presence of the two PL peaks has also been reported in several works.[1, 63-66]

Author Information

Corresponding authors

*E-mail: osbel.almora@fau.de, gematt@ethz.ch, garciag@uji.es

ORCID

O. Almora	https://orcid.org/0000-0002-2523-0203
G.J. Matt	https://orcid.org/0000-0002-1138-3671
A. These	https://orcid.org/0000-0001-6058-1191
A. Kanak	https://orcid.org/0000-0001-9238-4029
I. Levchuk	https://orcid.org/0000-0003-0644-2283
S. Shrestha	https://orcid.org/0000-0002-3606-7624
A. Osvet	https://orcid.org/0000-0001-9098-9171
C.J. Brabec	https://orcid.org/0000-0002-9440-0253
G. Garcia-Belmonte	https://orcid.org/0000-0002-0172-6175

Acknowledgments

We acknowledge the funding from the European Union's Horizon 2020 research and innovation program under the Photonics Public Private Partnership (www.photonics21.org) with the project PEROXIS under the grant agreement N° 871336. A.T. gratefully acknowledges the funding of the Erlangen Graduate School in Advanced Optical Technologies (SAOT) by the Bavarian State Ministry for Science and Art and financial support from the Deutsche Forschungsgemeinschaft (DFG) under MA 6617/1-1. Funding for open access charge: CRUE-Universitat Jaume I.

References

(1) Stoumpos, C. C.; Malliakas, C. D.; Peters, J. A.; Liu, Z.; Sebastian, M.; Im, J.; Chasapis, T. C.; Wibowo, A. C.; Chung, D. Y.; Freeman, A. J.; et al. Crystal Growth of the Perovskite Semiconductor $CsPbBr_3$: A New Material for High-Energy Radiation Detection. *Cryst. Growth Des.* **2013**, *13*, 2722-2727.

(2) Rakita, Y.; Kedem, N.; Gupta, S.; Sadhanala, A.; Kalchenko, V.; Böhm, M. L.; Kulbak, M.; Friend, R. H.; Cahen, D.; Hodes, G. Low-Temperature Solution-Grown $CsPbBr_3$ Single Crystals and Their Characterization. *Cryst. Growth Des.* **2016**, *16*, 5717-5725.

(3) Saidaminov, M. I.; Haque, M. A.; Almutlaq, J.; Sarmah, S.; Miao, X.-H.; Begum, R.; Zhumekenov, A. A.; Dursun, I.; Cho, N.; Murali, B.; et al. Inorganic Lead Halide Perovskite Single Crystals: Phase-Selective Low-Temperature Growth, Carrier Transport Properties, and Self-Powered Photodetection. *Adv. Opt. Mater.* **2017**, *5*, 1600704.

(4) Song, J.; Cui, Q.; Li, J.; Xu, J.; Wang, Y.; Xu, L.; Xue, J.; Dong, Y.; Tian, T.; Sun, H.; et al. Ultralarge All-Inorganic Perovskite Bulk Single Crystal for High-Performance Visible–Infrared Dual-Modal Photodetectors. *Adv. Opt. Mater.* **2017**, *5*, 1700157.

(5) Ding, J.; Du, S.; Zuo, Z.; Zhao, Y.; Cui, H.; Zhan, X. High Detectivity and Rapid Response in Perovskite $CsPbBr_3$ Single-Crystal Photodetector. *J. Phys. Chem. C* **2017**, *121*, 4917-4923.

(6) Cha, J.-H.; Han, J. H.; Yin, W.; Park, C.; Park, Y.; Ahn, T. K.; Cho, J. H.; Jung, D.-Y. Photoresponse of $CsPbBr_3$ and Cs_4PbBr_6 Perovskite Single Crystals. *J. Phys. Chem. Lett.* **2017**, *8*, 565-570.

(7) Chen, J.; Morrow, D. J.; Fu, Y.; Zheng, W.; Zhao, Y.; Dang, L.; Stolt, M. J.; Kohler, D. D.; Wang, X.; Czech, K. J.; et al. Single-Crystal Thin Films of Cesium Lead Bromide Perovskite Epitaxially Grown on Metal Oxide Perovskite ($SrTiO_3$). *J. Am. Chem. Soc.* **2017**, *139*, 13525-13532.

(8) He, Y.; Matei, L.; Jung, H. J.; McCall, K. M.; Chen, M.; Stoumpos, C. C.; Liu, Z.; Peters, J. A.; Chung, D. Y.; Wessels, B. W.; et al. High spectral resolution of gamma-rays at room temperature by perovskite $CsPbBr_3$ single crystals. *Nat. Commun.* **2018**, *9*, 1609.

(9) Zhang, M.; Zheng, Z.; Fu, Q.; Guo, P.; Zhang, S.; Chen, C.; Chen, H.; Wang, M.; Luo, W.; Tian, Y. Determination of Defect Levels in Melt-Grown All-Inorganic Perovskite $CsPbBr_3$ Crystals by Thermally Stimulated Current Spectra. *J. Phys. Chem. C* **2018**, *122*, 10309-10315.

(10) Zhang, M.; Zheng, Z.; Fu, Q.; Chen, Z.; He, J.; Zhang, S.; Chen, C.; Luo, W. Synthesis and single crystal growth of perovskite semiconductor $CsPbBr_3$. *J. Crystal Growth* **2018**, *484*, 37-42.

(11) Chen, C.; Fu, Q.; Guo, P.; Chen, H.; Wang, M.; Luo, W.; Zheng, Z. Ionic Transport Characteristics Of Large-Size $CsPbBr_3$ Single Crystals. *Mater. Res. Express* **2019**, *6*, 115808.

(12) Li, J.; Du, X.; Niu, G.; Xie, H.; Chen, Y.; Yuan, Y.; Gao, Y.; Xiao, H.; Tang, J.; Pan, A.; et al. Rubidium Doping to Enhance Carrier Transport in $CsPbBr_3$ Single Crystals for High-Performance X-Ray Detection. *ACS Appl. Mater. Interfaces* **2020**, *12*, 989-996.

(13) Wang, K.; Jing, L.; Yao, Q.; Zhang, J.; Cheng, X.; Yuan, Y.; Shang, C.; Ding, J.; Zhou, T.; Sun, H.; et al. Highly In-Plane Polarization-Sensitive Photodetection in $CsPbBr_3$ Single Crystal. *J. Phys. Chem. Lett.* **2021**, *12*, 1904-1910.

(14) Bagley, B. G. The field dependent mobility of localized electronic carriers. *Solid State Commun.* **1970**, *8*, 345-348.

(15) Natali, D.; Sampietro, M. Field-dependent mobility from space-charge-limited current–voltage curves. *J. Appl. Phys.* **2002**, *92*, 5310-5318.

(16) Kemp, D.; De Souza, R. A. Nonlinear Ion Mobility at High Electric Field Strengths in the Perovskites $SrTiO_3$ and $CH_3NH_3PbI_3$. *Phys. Rev. Mater.* **2021**, *5*, 105401.

(17) Nath, R.; Perlman, M. M. Steady-state bulk trap-modulated hopping conduction in doped linear low-density polyethylene. *J. Appl. Phys.* **1989**, *65*, 4854-4858.

(18) Kumar, A.; Perlman, M. M. Steady-state conduction in high density polyethylene with field-dependent mobility. *J. Appl. Phys.* **1992**, *71*, 735-738.

(19) Mizutani, T.; Ieda, M. Carrier transport in high-density polyethylene. *J. Phys. D Appl. Phys.* **1979**, *12*, 291-296.

(20) Yu, Z. G.; Smith, D. L.; Saxena, A.; Martin, R. L.; Bishop, A. R. Molecular geometry fluctuations and field-dependent mobility in conjugated polymers. *Phys. Rev. B* **2001**, *63*, 085202.

(21) Pautmeier, L.; Richert, R.; Bässler, H. Poole-Frenkel Behavior of Charge Transport in Organic Solids with Off-Diagonal Disorder Studied by Monte Carlo Simulation. *Synth. Met.* **1990**, *37*, 271-281.

(22) Bässler, H. Charge Transport in Disordered Organic Photoconductors a Monte Carlo Simulation Study. *Phys. Status Solidi B* **1993**, *175*, 15-56.

(23) Dunlap, D. H.; Parris, P. E.; Kenkre, V. M. Charge-Dipole Model for the Universal Field Dependence of Mobilities in Molecularly Doped Polymers. *Phys. Rev. Lett.* **1996**, *77*, 542-545.

(24) Montero, J. M.; Bisquert, J. Trap origin of field-dependent mobility of the carrier transport in organic layers. *Solid-State Electron.* **2011**, *55*, 1-4.

(25) Montero, J. M.; Bisquert, J.; Garcia-Belmonte, G.; Bolink, H. J.; Barea, E. M.

Interpretation of capacitance spectra and transit times of single carrier space-charge limited transport in organic layers with field-dependent mobility. *Phys. Status Solidi A* **2007**, *204*, 2402-2410.

(26) Bange, S.; Schubert, M.; Neher, D. Charge mobility determination by current extraction under linear increasing voltages: Case of nonequilibrium charges and field-dependent mobilities. *Phys. Rev. B* **2010**, *81*, 035209.

(27) Kreouzis, T.; Poplavskyy, D.; Tuladhar, S. M.; Campoy-Quiles, M.; Nelson, J.; Campbell, A. J.; Bradley, D. D. C. Temperature and field dependence of hole mobility in poly(9,9-dioctylfluorene). *Phys. Rev. B* **2006**, *73*, 235201.

(28) Campbell, I. H.; Smith, D. L.; Neef, C. J.; Ferraris, J. P. Consistent time-of-flight mobility measurements and polymer light-emitting diode current–voltage characteristics. *Appl. Phys. Lett.* **1999**, *74*, 2809-2811.

(29) Schein, L. B.; Peled, A.; Glatz, D. The electric field dependence of the mobility in molecularly doped polymers. *J. Appl. Phys.* **1989**, *66*, 686-692.

(30) Tripathi, D. C.; Rao, K. S.; Kumar, S.; Mohapatra, Y. N. Impact of device structure on field dependence of carrier mobility. *Synth. Met.* **2021**, *278*, 116835.

(31) Ziari, Z.; Sahli, S.; Bellel, A. Mobility Dependence on Electric Field in Low Density Polyethylene (LDPE). *Moroccan J. Condens. Matter* **2011**, *12*, 223.

(32) Kasap, S. O.; Juhasz, C. Time-of-flight drift mobility measurements on chlorine-doped amorphous selenium films. *J. Phys. D Appl. Phys.* **1985**, *18*, 703-720.

(33) Hirao, A.; Nishizawa, H.; Sugiuchi, M. Diffusion and Drift of Charge Carriers in Molecularly Doped Polymers. *Phys. Rev. Lett.* **1995**, *75*, 1787-1790.

(34) Augelli, V.; Manfredotti, C.; Murri, R.; Vasanelli, L. Hall-mobility anisotropy in GaSe. *Phys. Rev. B* **1978**, *17*, 3221-3226.

(35) Sonnonstine, T. J.; Perlman, M. M. Surface-potential decay in insulators with field-dependent mobility and injection efficiency. *J. Appl. Phys.* **1975**, *46*, 3975-3981.

(36) Wintle, H. J. Surface-Charge Decay in Insulators with Nonconstant Mobility and with Deep Trapping. *J. Appl. Phys.* **1972**, *43*, 2927-2930.

(37) Stratton, R.; Fröhlich, H. The influence of interelectronic collisions on conduction and breakdown in polar crystals. *Proc. R. Soc. London, Ser. A* **1958**, *246*, 406-422.

(38) Lu, N.; Li, L.; Banerjee, W.; Sun, P.; Gao, N.; Liu, M. Charge Carrier Hopping Transport Based On Marcus Theory and Variable-Range Hopping Theory in Organic Semiconductors. *J. Appl. Phys.* **2015**, *118*, 045701.

(39) Kadashchuk, A.; Janneck, R.; Tong, F.; Fishchuk, I. I.; Mityashin, A.; Pavlica, E.; Köhler, A.; Heremans, P.; Rolin, C.; Bratina, G.; et al. Negative field-dependent charge

mobility in crystalline organic semiconductors with delocalized transport. *Chem. Papers* **2018**, *72*, 1685-1695.

(40) Kadashchuk, A.; Tong, F.; Janneck, R.; Fishchuk, I. I.; Mityashin, A.; Pavlica, E.; Köhler, A.; Heremans, P.; Rolin, C.; Bratina, G.; et al. Role of transport band edge variation on delocalized charge transport in high-mobility crystalline organic semiconductors. *Phys. Rev. B* **2017**, *96*, 125202.

(41) Oelerich, J. O.; Nenashev, A. V.; Dvurechenskii, A. V.; Gebhard, F.; Baranovskii, S. D. Field Dependence of Hopping Mobility: Lattice Models against Spatial Disorder. *Phys. Rev. B* **2017**, *96*, 195208.

(42) Gailberger, M.; Bässler, H. dc and transient photoconductivity of poly(2-phenyl-1,4-phenylenevinylene). *Phys. Rev. B* **1991**, *44*, 8643-8651.

(43) Bouhassoune, M.; Mensfoort, S. L. M. v.; Bobbert, P. A.; Coehoorn, R. Carrier-Density and Field-Dependent Charge-Carrier Mobility in Organic Semiconductors with Correlated Gaussian Disorder. *Org. Electron.* **2009**, *10*, 437-445.

(44) Bhattarai, G.; Caruso, A. N.; Paquette, M. M. Steady-state space-charge-limited current analysis of mobility with negative electric field dependence. *J. Appl. Phys.* **2018**, *124*, 045701.

(45) Mozer, A. J.; Sariciftci, N. S. Negative electric field dependence of charge carrier drift mobility in conjugated, semiconducting polymers. *Chem. Phys. Lett.* **2004**, *389*, 438-442.

(46) Raj Mohan, S.; Joshi, M. P.; Singh, M. P. Negative electric field dependence of mobility in TPD doped Polystyrene. *Chem. Phys. Lett.* **2009**, *470*, 279-284.

(47) Tripathi, A. K.; Ashish; Mohapatra, Y. N. Mobility with negative coefficient in Poole–Frenkel field dependence in conjugated polymers: Role of injected hot electrons. *Org. Electron.* **2010**, *11*, 1753-1758.

(48) Juška, G.; Genevičius, K.; Arlauskas, K.; Österbacka, R.; Stubb, H. Charge transport at low electric fields in π-conjugated polymers. *Phys. Rev. B* **2002**, *65*, 233208.

(49) Raj Mohan, S.; Singh, M. P.; Joshi, M. P. Negative field dependence of mobility in disordered organic thin films due to non-equilibrium charge transport. *Org. Electron.* **2010**, *11*, 1642-1648.

(50) Singh, W. J.; Singh, K. J.; Ramesh, K. P.; Devi, K. N. Dopant induced anomalous field dependent mobility behavior of poly(3-octylthiophene) devices. *Synth. Met.* **2021**, *279*, 116855.

(51) Raj Mohan, S.; Joshi, M. P. Field dependence of hole mobility in TPD-doped polystyrene. *Solid State Commun.* **2006**, *139*, 181-185.

(52) Gambino, S.; Samuel, I. D. W.; Barcena, H.; Burn, P. L. Electric field and

temperature dependence of the hole mobility in a bis-fluorene cored dendrimer. *Org. Electron.* **2008**, *9*, 220-226.

(53) Kažukauskas, V.; Pranaitis, M.; Čyras, V.; Sicot, L.; Kajzar, F. Negative mobility dependence on electric field in poly(3-alkylthiophenes) evidenced by the charge extraction by linearly increasing voltage method. *Thin Solid Films* **2008**, *516*, 8988-8992.

(54) Peled, A.; Schein, L. B. Hole mobilities that decrease with increasing electric fields in a molecularly doped polymer. *Chem. Phys. Lett.* **1988**, *153*, 422-424.

(55) Chen, Z.; Ma, D. Effects of doped dye on the charge carrier injection, transport, and electroluminescent performance in polymeric light-emitting diodes. *J. Appl. Phys.* **2007**, *102*, 024510.

(56) Kažukauskas, V.; Pranaitis, M.; Sicot, L.; Kajzar, F. Negative Mobility Dependence in Different Regioregular Polythiophenes Revealed by the Charge Extraction by Linearly Increasing Voltage Method. *Mol. Cryst. Liq. Cryst.* **2006**, *447*, 141/[459]-153/[471].

(57) Huang, J.; Li, G.; Yang, Y. Influence of composition and heat-treatment on the charge transport properties of poly(3-hexylthiophene) and [6,6]-phenyl C61-butyric acid methyl ester blends. *Appl. Phys. Lett.* **2005**, *87*, 112105.

(58) Gartstein, Y. N.; Conwell, E. M. High-Field Hopping Mobility of Polarons in Disordered Molecular Solids. A Monte Carlo Study. *Chem. Phys. Lett.* **1994**, *217*, 41-47.

(59) Zhang, P.; Zhang, G.; Liu, L.; Ju, D.; Zhang, L.; Cheng, K.; Tao, X. Anisotropic Optoelectronic Properties of Melt-Grown Bulk $CsPbBr_3$ Single Crystal. *J. Phys. Chem. Lett.* **2018**, *9*, 5040-5046.

(60) Fan, Z.; Liu, J.; Zuo, W.; Liu, G.; He, X.; Luo, K.; Ye, Q.; Liao, C. Solution-Processed $MAPbBr_3$ and $CsPbBr_3$ Single-Crystal Detectors with Improved X-Ray Sensitivity via Interfacial Engineering. *Phys. Status Solidi A* **2020**, *217*, 2000104.

(61) He, H.; Ma, E.; Chen, X.; Yang, D.; Chen, B.; Qian, G. Single Crystal Perovskite Microplate for High-Order Multiphoton Excitation. *Small Methods* **2019**, *3*, 1900396.

(62) Zhao, L.; Gao, Y.; Su, M.; Shang, Q.; Liu, Z.; Li, Q.; Wei, Q.; Li, M.; Fu, L.; Zhong, Y.; et al. Vapor-Phase Incommensurate Heteroepitaxy of Oriented Single-Crystal CsPbBr3 on GaN: Toward Integrated Optoelectronic Applications. *ACS Nano* **2019**, *13*, 10085-10094.

(63) Kim, D.; Ryu, H.; Lim, S. Y.; McCall, K. M.; Park, J.; Kim, S.; Kim, T. J.; Kim, J.; Kim, Y. S.; Kanatzidis, M. G.; et al. On the Origin of Room-Temperature Amplified Spontaneous Emission in $CsPbBr_3$ Single Crystals. *Chem. Mater.* **2021**, *33*, 7185-7193.

(64) Shibata, K.; Yan, J.; Hazama, Y.; Chen, S.; Akiyama, H. Exciton Localization and Enhancement of the Exciton–LO Phonon Interaction in a $CsPbBr_3$ Single Crystal. *J. Phys. Chem. C* **2020**, *124*, 18257-18263.

(65) Sebastian, M.; Peters, J. A.; Stoumpos, C. C.; Im, J.; Kostina, S. S.; Liu, Z.; Kanatzidis, M. G.; Freeman, A. J.; Wessels, B. W. Excitonic Emissions and Above-Band-Gap Luminescence in the Single-Crystal Perovskite Semiconductors $CsPbBr_3$ and $CsPbCl_3$. *Phys. Rev. B* **2015**, *92*, 235210.

(66) Zhang, H.; Liu, X.; Dong, J.; Yu, H.; Zhou, C.; Zhang, B.; Xu, Y.; Jie, W. Centimeter-Sized Inorganic Lead Halide Perovskite CsPbBr3 Crystals Grown by an Improved Solution Method. *Cryst. Growth Des.* **2017**, *17*, 6426-6431.
